# Prevalence and correlates of suicidal ideation and suicide attempts in preadolescent children: A US population-based study

**DOI:** 10.1038/s41398-021-01593-3

**Published:** 2021-09-22

**Authors:** Hannah R. Lawrence, Taylor A. Burke, Ana E. Sheehan, Brianna Pastro, Rachel Y. Levin, Rachel F. L. Walsh, Alexandra H. Bettis, Richard T. Liu

**Affiliations:** 1 Department of Psychiatry, McLean Hospital, Belmont, CA USA; 2 Harvard Medical School, Boston, MA USA; 3 Department of Psychiatry, Massachusetts General Hospital, Boston, MA USA; 4 Department of Psychological and Brain Sciences, University of Delaware, Newark, DE USA; 5 Department of Psychology, Temple University, Philadelphia, PA USA; 6 Department of Psychiatry and Behavioral Sciences, Vanderbilt University Medical Center, Nashville, TN USA

**Keywords:** Scientific community, Depression, Psychiatric disorders, Human behaviour

## Abstract

The present study evaluated sociodemographic and diagnostic predictors of suicidal ideation and attempts in a nationally representative sample of preadolescent youth enrolled in the Adolescent Brain Cognitive Development Study. Rates and predictors of psychiatric treatment utilization among suicidal youth also were examined. Eleven thousand eight hundred and seventy-five 9- and 10-year-old children residing in the United States were assessed. Children and their parents/guardians provided reports of children’s lifetime history of suicidal ideation, suicide attempts, and psychiatric disorders. Parents also reported on sociodemographic characteristics and mental health service utilization. Multivariate logistic regression analyses were employed to evaluate sociodemographic and diagnostic correlates of suicidal ideation, suicide attempts among youth with suicidal ideation, and treatment utilization among youth with suicidal ideation and suicide attempts. Lifetime prevalence rates were 14.33% for suicidal ideation and 1.26% for suicide attempts. Youth who identified as male, a sexual minority, or multiracial had greater odds of suicidal ideation, and sexual minority youth and youth with a low family income had greater odds of suicide attempts. Comorbid psychopathology was associated with higher odds of both suicidal ideation and suicide attempts. In youth, 34.59% who have suicidal ideation and 54.82% who had attempted suicide received psychiatric treatment. Treatment utilization among suicidal youth was lower among those who identified as female, Black, and Hispanic. Suicidal ideation and attempts among preadolescent children are concerningly high and targeted assessment and preventative efforts are needed, especially for males, racial, ethnic, and sexual minority youth, and those youth experiencing comorbidity.

## Predictors of suicidal ideation and suicide attempts in preadolescent children: A US population-based study

Suicide is a leading cause of death among youth [1], but strikingly little is known regarding predictors of suicide risk in preadolescent children. What research has been done suggests that rates of suicide attempts and deaths in preadolescent children have not decreased in recent years and may even be on the rise [1–4]. In recognition of this concern, the National Institute of Mental Health has designated reducing childhood suicide as a priority [5] and recently assembled a panel for 2021 focusing specifically on this issue.

Although prior nationally representative studies of suicide have made strides in documenting trends in rates of suicidal ideation, suicidal behavior, and death by suicide among youth, they have largely been primarily conducted with adolescents [6–8], examined non-US samples [9, 10], or focused solely on suicide deaths in children [2–4, 11]. Suicidal ideation and suicide attempts are clinically relevant in their own right given that experiencing suicidal thoughts and behaviors in childhood is associated concurrently and prospectively with morbidity [9, 12, 13]. In addition, earlier onset of suicidal ideation and behavior may be associated with amplified risk for poor outcomes [13], further justifying the need for nationally representative data on suicide risk in preadolescent children to accurately characterize the scope of this issue.

What non-nationally representative data do exist on factors associated with preadolescent suicide risk suggest that economic disadvantage [14, 15] and female [15] sex may be associated with suicidal ideation and/or behavior, whereas differences based on race are not typically found [16–18]. Critically, however, findings have been notably mixed (e.g., O’Leary and colleagues found no significant differences in preadolescent suicidal ideation by sex [16]) and sample sizes small. Prior analysis of Adolescent Brain Cognitive Development (ABCD) study data found sex differences in suicide risk such that there were higher rates of suicidal ideation but not suicide attempts among males compared with females and identified no significant differences in suicidal ideation or attempts by race and ethnicity [19]. Importantly, however, racial and ethnic identities were treated as categories within a single dimension and suicidal ideation was conflated with attempts, preventing conclusions being reached regarding factors uniquely associated with suicide ideation or with attempts among youth with suicidal ideation.

In terms of potential diagnostic predictors, suicidal preadolescent youth were significantly more likely to have a psychiatric disorder and especially multiple psychiatric disorders compared to nonsuicidal preadolescent youth in the Great Smoky Mountains Study [14]. Although depression has been the most consistently studied diagnostic predictor of preadolescent suicide, anxiety disorders, disruptive behavior disorders, and attention-deficit/hyperactivity disorder (ADHD) also may be associated with suicidal ideation and/or behavior in this age group [14, 18]. Again, however, no research has evaluated diagnostic predictors of suicidal ideation and attempts in nationally representative samples of preadolescent youth, and it remains unknown which psychiatric diagnoses are associated with suicidal ideation only or suicide attempts among youth with suicidal ideation.

Data also are needed on rates of treatment utilization among preadolescent children who have experienced suicidal ideation or attempts. Population-based studies with adolescent samples find that among adolescents who have experienced suicidal ideation or who have made a suicide attempt, many do not have contact with mental health providers [20, 21]. It is not yet known, however, what proportion of preadolescent children with a history of suicidal ideation and/or attempts engage with mental health services, nor what factors predict which children receive treatment. Given that delay in receipt of treatment could result in youth not being regularly assessed for imminent suicide risk and learning strategies to reduce this risk, it is vitally important to identify which preadolescent children are not accessing treatment.

The current paper extends prior work, presenting nationally representative data on predictors of suicidal ideation and attempts in preadolescent children in the US from the ABCD Study. Prior studies on suicide risk in the ABCD Study have reported prevalence rates of self-injurious thoughts and behaviors (SITBs), interinformant agreement on reporting of SITBs, and tested whether familial characteristics and dimensions of internalizing and externalizing symptoms predict risk [19, 22]. Here, we build on these studies by evaluating sociodemographic and diagnostic predictors of suicidal ideation and attempts, and by examining predictors of treatment utilization among youth with suicidal ideation and suicide attempts.

## Method

### Sample

The ABCD Study is a longitudinal, multimethod, multi-informant study following children in the US over 10 years to examine mental health trajectories from childhood through adulthood. The present study used the National Data Archive ABCD 2.01 baseline dataset [23], which was collected between 2016 and 2018. The ABCD Study surveyed 11,875 preadolescent children currently residing in the US, all of whom were 9 or 10 years of age at enrollment. Participants were recruited based on age, sex, race, socioeconomic status (SES), and urbanicity for the purpose of matching SES variation in the US. Participants were enrolled at 22 research sites. For each child, one parent or guardian provided informed consent and information about themselves and their child. The design, procedure, and weighting procedures of this study have been reported elsewhere [24–26]. All study procedures were approved by The University of California, San Diego institutional review board (IRB), which serves as the central IRB for the study. Children in the weighted sample reflect the larger population of U.S. 9- and 10-year-olds in terms of sociodemographic and geographic factors.

### Measures

#### Suicidal ideation and behavior

Child and parent self-reports of current and past suicidal ideation and behavior were obtained using the computerized version of the Kiddie Schedule for Affective Disorders and Schizophrenia–Present and Lifetime Version for DSM-5 (K-SADS-PL DSM-5) [27]. Items included those assessing passive and active suicidal ideation and suicide attempts. In line with standard clinical practice [28], the child was coded as having lifetime suicidal ideation or having made a lifetime suicide attempt if either the parent or the child endorsed any of the relevant items either currently (i.e., in the past 2 weeks) or in the past^1^. Variables were then computed to (1) compare children who had only suicidal ideation versus children who reported neither suicidal ideation nor suicide attempts and (2) to compare children who had and had not experienced a suicide attempt among those children with suicidal ideation.

#### Sociodemographic variables

Each child’s parent or guardian completed a demographic questionnaire, which asked about the child’s sex, racial and ethnic identities, family income, parent/guardian education level, and parent/guardian marital status. Youth also reported on their sexual orientation. For the purposes of retaining sufficient power for analyses, categories for race were collapsed into “White,” “Black,” “Multiracial,” and “Other race,” sexual orientation was collapsed into “Gay or bisexual,” “Not gay or bisexual,” and “Did not understand the question,” family income categories were collapsed into five categories ranging from “Less than $25,000” to “$100,000 and greater”^2^, parental education was collapsed into four categories ranging from “Less than high school” to “College graduate,” and parental marital status was collapsed into “Not married” and “married.”^3^

#### DSM-5 mental health disorders and psychiatric treatment utilization

The K-SADS-PL DSM-5 also was used to obtain information on whether each child met lifetime DSM-5 criteria for mental health disorders. Disorders assessed included: major depressive disorder (MDD), separation anxiety disorder, social anxiety disorder, specific phobia, generalized anxiety disorder (GAD), obsessive compulsive disorder (OCD), post-traumatic stress disorder (PTSD), conduct disorder, oppositional defiant disorder (ODD), and attention-deficit hyperactivity disorder (ADHD). Given their extremely low prevalence rates, all eating disorders were collapsed into one category, as were any psychotic disorder. Again, in line with standard clinical practice, a child was coded as having met lifetime criteria for a psychiatric diagnosis if they met criteria based on parent report, child report, or both either currently (i.e., in the past 2 weeks) or in the past.

Parents also reported whether their child had ever received mental health treatment. Specifically, they responded to the question, “Has your child ever received mental health or substance abuse services?” (yes, no, not sure^4^). Although parents also indicted which types of mental health treatment their child received (outpatient, partial hospital, inpatient, outpatient substance abuse, partial hospital for substance abuse, inpatient for substance abuse, psychotherapy, medication management, other), some response categories were too infrequently endorsed for meaningful analyses.

### Statistical analyses

Cross-tabulations were used to estimate the lifetime prevalence of suicidal ideation, suicide attempts, DSM-5 mental health disorders, and treatment utilization. Cross-tabulations also were conducted to produce lifetime prevalence estimates of suicidal ideation and suicide attempts among youth with various sociodemographic characteristics and mental health diagnoses and to estimate prevalence of treatment utilization among youth with suicidal ideation and suicide attempts. A series of bivariate logistic regression analyses were then conducted to test sociodemographic and diagnostic predictors of suicidal ideation, suicide attempts, and treatment utilization among youth with suicidal ideation or suicide attempts. The Benjamini–Hochberg procedure was applied to correct for multiple comparisons in bivariate analyses. Multivariate logistic regression analyses followed in which all sociodemographic factors were included in one model, any diagnosis versus no diagnosis was included in a second model, number of diagnoses (none, single, two, or more) was included in a third model, and all diagnostic factors were included in a fourth model. Sociodemographic factors were covaried in the latter three models. In all cases, the reference group was defined as the category hypothesized to be at lowest risk.

Dependent variables in separate models were suicidal ideation, suicide attempts, and treatment utilization. In the case of analyses with suicidal ideation as the criterion variable, youth with a lifetime history of suicide attempts were excluded to compare “pure” suicidal ideation versus no suicidal ideation^5^. In the case of analyses with suicide attempts as the criterion variable, lifetime history of suicide attempt versus no lifetime history of suicide attempt was compared among youth with suicidal ideation. Treatment utilization was defined as having a lifetime history of any mental health treatment. Results from all regression analyses are presented as odds ratios with 95% confidence intervals. SPSS 27.0 was used to conduct all analyses and weighting procedures were applied to accommodate the complex sampling frame of the survey and to generate nationally representative estimates.

## Results

In the full sample (unweighted *N* = 11,875), the total lifetime prevalence rates for suicidal ideation was 14.33% (SE = 0.37) and for suicide attempts was 1.26% (SE = 0.12). 3.62% (SE = 0.20) of the sample reported current suicidal ideation and 0.26% (SE = 0.06) reported a current suicide attempt. 13.15% (SE = 0.35) of youth reported a lifetime history of pure suicidal ideation. Among those with a lifetime history of suicidal ideation, 8.70% (SE = 0.82) reported a lifetime history of suicide attempt. Sociodemographic characteristics of the subsamples of youth with lifetime histories of pure suicidal ideation and suicide attempts are found in Table 1.

**Table 1:** Sociodemographic and clinical characteristics of the sample (unweighted *N* = 11875).

	Total sample	Pure suicidal ideation	Suicide attempts
	% (SE)	% (SE)	% (SE)
Sex			
Female	48.85 (0.52)	11.35 (0.49)	9.45 (1.33)
Male	51.15 (0.52)	15.24 (0.52)	8.20 (1.04)
Sexual orientation			
Gay or bisexual	1.33 (0.12)	34.14 (4.51)	17.61 (5.98)
Do not understand the question	24.57 (0.44)	13.01 (0.71)	9.17 (1.76)
Not gay or bisexual	74.10 (0.45)	13.15 (0.41)	8.11 (0.92)
Race			
Black	14.57 (0.34)	11.10 (0.77)	15.68 (2.52)
Multiracial	8.10 (0.25)	17.01 (1.22)	8.12 (2.10)
Other Race	10.20 (0.36)	13.25 (1.32)	7.64 (2.69)
White	67.14 (0.48)	13.40 (0.45)	7.54 (1.00)
Ethnicity			
Hispanic	24.20 (0.46)	12.10 (0.73)	11.08 (2.01)
Non-Hispanic	75.80 (0.46)	13.76 (0.42)	8.15 (0.90)
Family Income			
Less than $25,000	18.74 (0.45)	12.70 (0.91)	14.30 (2.54)
$25,000 to $49,999	20.37 (0.48)	14.36 (0.97)	13.29 (2.31)
$50,000 to $74,999	17.49 (0.44)	15.85 (1.03)	6.64 (1.81)
$75,000 through $99,999	13.40 (0.35)	12.84 (0.93)	5.64 (1.72)
$100,000 and greater	30.00 (0.45)	12.04 (0.53)	4.06 (0.86)
Parental education			
Less than high school	1.81 (0.15)	13.15 (2.92)	13.72 (7.89)
High school or GED	18.48 (0.42)	10.99 (0.79)	14.24 (2.63)
Some college	18.19 (0.41)	14.11 (0.89)	7.40 (1.73)
College graduate	61.52 (0.51)	13.86 (0.45)	7.55 (0.95)
Parental marital status			
Not married	38.82 (0.52)	15.03 (0.65)	11.26 (1.43)
Married	61.18 (0.52)	12.34 (0.42)	6.66 (0.93)
Diagnoses			
Any disorder	51.12 (0.52)	18.91 (0.58)	9.60 (1.03)
Number of diagnoses	24.99 (0.45)		
Single disorder	26.13 (0.46)	12.61 (0.69)	5.94 (1.40)
Two or more disorders	51.12 (0.52)	25.08 (0.92)	11.30 (1.35)
Disorder type			
MDD	5.63 (0.25)	43.67 (2.30)	11.53 (2.24)
Any anxiety disorder	38.33 (0.51)	18.10 (0.66)	10.95 (1.27)
Separation anxiety	9.25 (0.31)	24.48 (1.55)	8.74 (1.92)
Social anxiety	5.28 (0.23)	24.86 (2.04)	15.99 (3.32)
Specific phobia	27.53 (0.47)	16.59 (0.76)	11.88 (1.67)
GAD	5.13 (0.23)	35.48 (2.30)	12.52 (2.61)
OCD	9.83 (0.31)	21.09 (1.40)	11.71 (2.37)
PTSD	2.23 (0.16)	36.23 (3.67)	14.49 (4.26)
Any behavioral disorder	15.55 (0.38)	28.94 (1.24)	11.37 (1.64)
Conduct disorder	3.38 (0.19)	36.83 (2.91)	15.16 (3.56)
ODD	14.34 (0.37)	29.24 (1.29)	10.67 (1.65)
ADHD	21.35 (0.43)	23.14 (0.99)	10.23 (1.47)
Eating disorders	0.95 (0.10)	28.16 (5.17)	17.39 (7.12)
Psychosis	1.15 (0.12)	22.70 (4.55)	22.26 (6.90)

*ADHD* attention-deficit hyperactivity disorder, *GAD* generalized anxiety disorder, *GED* General Educational Development, *MDD* major depressive disorder, *OCD* obsessive compulsive disorder, *ODD* oppositional defiant disorder, *PTSD* post-traumatic stress disorder, Any anxiety disorder panic disorder, agoraphobia, separation anxiety disorder, social anxiety disorder, specific phobia, generalized anxiety disorder, obsessive compulsive disorder, or post-traumatic stress disorder, Any behavioral disorder = conduct disorder or oppositional defiant disorder.

Percent and standard error are weighted. Weighted prevalence rates of suicidal ideation and suicide attempts are presented for each predictor.

### Predictors of lifetime “pure” suicidal ideation

Results of the univariate and multivariate logistic regression analyses for sociodemographic predictors of pure suicidal ideation versus no suicidal ideation are presented in Table 2. When considering all sociodemographic variables together, female sex was associated with decreased odds of suicidal ideation (OR = 0.71, 95% CI 0.62–0.81), as was Hispanic ethnicity (OR = 0.73, 95% CI 0.60–0.87), identifying as Black (OR = 0.77, 95% CI 0.62–0.95), and having parents with a high school or GED level education (OR = 0.78, 95% CI 0.62–0.97). Sociodemographic characteristics associated with increased odds of suicidal ideation included identifying as a sexual minority (OR = 3.81, 95% CI 2.49–5.83), multiracial (OR = 1.39, 95% CI 1.13–1.70), having parents who were not married (OR = 1.30, 95% CI 1.10–1.53), and being in the second (OR = 1.29, 95% CI 1.03–1.63) or third (OR = 1.39, 95% CI 1.15–1.69) quintile for family income.

**Table 2:** Sociodemographic predictors of suicidal ideation and suicide attempts.

	Pure Suicidal Ideation^a^				Suicide Attempts^b^			
	Univariate		Multivariate		Univariate		Multivariate	
	OR (95% CI)	*p*	OR (95% CI)	*p*	OR (95% CI)	*p*	OR (95% CI)	*p*
Sex								
Female	0.71 (0.63–0.81)	<0.001	0.71 (0.62–0.81)	<0.001	1.17 (0.78–1.76)	0.45	0.96 (0.62–1.51)	0.87
Male (reference)	1.00		1.00		1.00		1.00	
Sexual orientation								
Gay or bisexual	3.42 (2.29–5.11)	<0.001	3.81 (2.49–5.83)	<0.001	2.42 (1.04–5.63)	0.04^c^	2.55 (1.02–6.38)	<0.05
Do not understand the question	0.99 (0.86–1.14)	0.86	0.99 (0.85–1.15)	0.86	1.14 (0.71–1.85)	0.58	1.40 (0.83–2.37)	0.20
Not gay or bisexual (reference)	1.00		1.00		1.00		1.00	
Race								
Black	0.81 (0.68–0.96)	0.01	0.77 (0.62–0.95)	0.02	2.28 (1.43–3.64)	<0.01	1.69 (0.90–3.18)	0.10
Multiracial	1.32 (1.10–1.60)	<0.01	1.39 (1.13–1.70)	<0.01	1.08 (0.58–2.01)	0.80	0.92 (0.46–1.84)	0.82
Other race	0.99 (0.78–1.25)	0.92	1.16 (0.89–1.52)	0.28	1.01 (0.46–2.26)	0.97	0.92 (0.37–2.28)	0.86
White (reference)	1.00		1.00		1.00		1.00	
Ethnicity								
Hispanic	0.86 (0.74–1.00)	0.06	0.73 (0.60–0.87)	<0.01	1.40 (0.88–2.23)	0.15	1.46 (0.82–2.58)	0.20
Non-Hispanic (reference)	0.88		1.00		1.00		1.00	
Family income								
Less than $25,000	1.06 (0.88–1.28)	0.52	1.18 (0.91–1.54)	0.21	3.94 (2.18–7.13)	<0.001	2.86 (1.31–6.26)	<0.01
$25,000–$49,999	1.23 (1.02–1.47)	0.03^c^	1.29 (1.03–1.63)	0.03	3.62 (2.02–6.49)	<0.001	3.08 (1.62–5.86)	<0.001
$50,000–$74,999	1.38 (1.15–1.65)	<0.001	1.39 (1.15–1.69)	<0.01	1.68 (0.82–3.45)	0.16	1.56 (0.74–3.33)	0.25
$75,000 through $99,999	1.08 (0.89–1.30)	0.45	1.11 (0.91–1.35)	0.30	1.41 (0.66–3.04)	0.38	1.32 (0.60–2.90)	0.50
$100,000 and greater (reference)	1.00		1.00		1.00		1.00	
Parental education								
Less than high school	0.94 (0.57–1.56)	0.81	1.15 (0.57–2.29)	0.70	1.95 (0.51–7.39)	0.33	–	–
High school or GED	0.77 (0.64–0.92)	<0.01	0.78 (0.62–0.97)	0.02	2.03 (1.23–3.35)	<0.01	1.14 (0.60–2.17)	0.70
Some college	1.02 (0.87–1.20)	0.81	0.90 (0.75–1.09)	0.27	0.98 (0.56–1.72)	0.94	0.64 (0.33–1.23)	0.18
College graduate (reference)	1.00		1.00		1.00		1.00	
Parental marital status								
Not married	1.26 (1.11–1.42)	<0.001	1.30 (1.10–1.53)	<0.01	1.78 (1.18–2.67)	<0.01	1.21 (0.70–2.09)	0.50
Married (reference)	1.00		1.00		1.00		1.00	

*CI* confidence interval, *GED* General Educational Development, *OR* odds ratio.

^a^Analyses conducted predicted suicidal ideation among youth with no suicide attempt history (unweighted *n* = 11708).

^b^Analyses conducted predicted suicide attempts among youth with suicidal ideation (unweighted *n* = 1668).

^c^Not significant after Benjamini–Hochberg correction applied.

Multivariate analyses controlled for all other sociodemographic factors.

Results of the univariate and multivariate logistic regression analyses for diagnostic predictors of pure suicidal ideation versus no suicidal ideation are presented in Table 3. Having any psychiatric condition was associated with greater odds of suicidal ideation (OR = 2.67, 95% CI 2.32–3.08), with the odds of suicidal ideation increasing when youth experienced two or more disorders (OR = 3.79, 95% CI 3.24–4.43). In the multivariate analysis controlling for sociodemographic variables, the following disorders emerged as significant predictors: MDD (OR = 4.47, 95% CI 3.54–5.64), GAD (OR = 1.85, 95% CI 1.41–2.44), conduct disorder (OR = 1.78, 95% CI 1.28–2.47), ODD (OR = 2.00, 95% CI 1.65–2.41), and ADHD (OR = 1.43, 95% CI 1.20–1.69).

**Table 3:** Diagnostic predictors of suicidal ideation and suicide attempts.

	Pure Suicidal Ideation^a^				Suicide Attempts^b^			
	Univariate		Multivariate		Univariate		Multivariate	
	OR (95% CI)	*p*	OR (95% CI)	*p*	OR (95% CI)	*p*	OR (95% CI)	*p*
Any disorder	2.73 (2.39–3.12)	<0.001	2.67 (2.32–3.08)	<0.001	1.56 (0.97–2.52)	0.07	1.85 (1.10–3.09)	0.02
Number of diagnoses								
Single disorder	1.69 (1.43–1.99)	<0.001	1.70 (1.43–2.03)	<0.001	0.93 (0.49–1.77)	0.82	1.21 (0.62–2.35)	0.59
Two or more disorders	3.92 (3.39–4.54)	<0.001	3.79 (3.24–4.43)	<0.001	1.87 (1.14–3.07)	0.01	2.17 (1.26–3.72)	<0.01
Disorder type								
MDD	5.89 (4.84–7.15)	<0.001	4.47 (3.54–5.64)	<0.001	1.48 (0.91–2.42)	0.11	1.04 (0.60–1.78)	0.90
Any anxiety disorder	1.89 (1.67–2.14)	<0.001			1.87 (1.23–2.84)	<0.01		
Separation anxiety	2.30 (1.93–2.74)	<0.001	1.15 (0.91–1.45)	0.23	1.01 (0.60–1.70)	0.98	0.47 (0.24–0.90)	0.02
Social anxiety	2.26 (1.81–2.83)	<0.001	1.19 (0.91–1.56)	0.21	2.23 (1.31–3.81)	<0.01	2.39 (1.25–4.58)	<0.01
Specific phobia	1.42 (1.24–1.62)	<0.001	0.98 (0.83–1.14)	0.76	1.79 (1.19–2.70)	<0.01	1.61 (0.98–2.65)	0.06
GAD	3.95 (3.21–4.86)	<0.001	1.85 (1.41–2.44)	<0.001	1.63 (0.97–2.73)	0.07	0.99 (0.51–0.93)	0.98
OCD	1.84 (1.54–2.20)	<0.001	1.02 (0.81–1.28)	0.89	1.50 (0.90–2.48)	0.12	1.10 (0.58–2.10)	0.77
PTSD	3.81 (2.77–5.23)	<0.001	1.25 (0.83–1.89)	0.29	1.87 (0.92–3.79)	0.08	1.25 (0.52–3.02)	0.62
Any behavioral disorder	3.40 (2.96–3.91)	<0.001			1.62 (1.07–2.45)	0.02^c^		
Conduct disorder	4.01 (3.12–5.17)	<0.001	1.78 (1.28–2.47)	<0.01	2.05 (1.14–3.68)	0.02	1.50 (0.67–3.36)	0.33
ODD	3.38 (2.94–3.90)	<0.001	2.00 (1.65–2.41)	<0.001	1.41 (0.92–2.16)	0.11	1.27 (0.71–2.26)	0.43
ADHD	2.47 (2.16–2.81)	<0.001	1.43 (1.20–1.69)	<0.001	1.35 (0.89–2.04)	0.16	1.01 (0.58–1.76)	0.97
Eating disorders	2.55 (1.54–4.22)	<0.001	0.93 (0.49–1.76)	0.83	2.27 (0.84–6.13)	0.11	1.91 (0.63–5.82)	0.25
Psychosis	1.91 (1.14–3.18)	0.01	0.98 (0.53–1.83)	0.95	3.13 (1.39–7.04)	<0.01	2.46 (0.85–7.08)	0.10

*CI* confidence interval, *GED* General Educational Development, *OR* odds ratio.

^a^Analyses conducted predicted pure suicidal ideation versus no suicidal ideation (unweighted *n* = 11708).

^b^Analyses conducted predicted suicide attempts versus no suicide attempts among youth with suicidal ideation (unweighted *n* = 1668).

^c^Not significant after Benjamini–Hochberg correction applied.

Multivariate analyses separated by blank rows represent separate models, each of which covaried all sociodemographic factors. The first model examined whether having any diagnosis predicted suicidal ideation and suicide attempts, the second assessed number of diagnoses (none, single disorder, two, or more disorders) as a predictor, and the third assessed each disorder as a predictor controlling for all other diagnoses. Only individual diagnoses were included in multivariate models to avoid overlap between individual diagnoses and grouped diagnoses (i.e., any anxiety disorder and any behavioral disorder).

### Predictors of lifetime suicide attempts

Sociodemographic predictors of lifetime history of suicide attempts versus no suicide attempts among youth with suicidal ideation were examined in a series of univariate and multivariate logistic regression analyses (Table 2). In a multivariate model accounting for all sociodemographic factors, the only characteristics that were significant predictors of suicide attempts were sexual minority status and family income. Identifying as gay or bisexual (OR = 2.55, 95% CI 1.02–6.38) and being in the first (OR = 2.86, 95% CI 1.31–6.26) or second (OR = 3.08, 95% CI 1.62–5.86) quintile of family income were associated with increased odds of suicide attempts.

Similarly, univariate and multivariate logistic regressions were used to determine diagnostic predictors of lifetime history of suicide attempts versus no suicide attempts among youth with suicidal ideation (Table 3). At the multivariate level, having a single disorder did not differentiate youth with suicide attempts from those with only suicidal ideation. However, having two or more psychiatric diagnoses significantly increased the odds of suicide attempts (OR = 2.17, 95% CI 1.26–3.72). When accounting for all diagnoses and controlling for sociodemographic variables in a multivariate analysis, only social anxiety (OR = 2.39, 95% CI 1.25–4.58) remained a significant predictor of suicide attempts, whereas separation anxiety was associated with lower odds of suicide attempts (OR = 0.47, 95% CI 0.24–0.90).

### Predictors of psychiatric treatment utilization

Lifetime history of suicidal ideation (OR = 3.48, 95% CI 3.03–3.99) and suicide attempts (OR = 2.30, 95% CI 1.53–3.45) predicted higher odds of receiving mental health treatment. Of youth with pure suicidal ideation, 34.59% (SE = 1.39) received treatment. Among youth with suicidal ideation, 54.82% (SE = 4.91) of youth who had a lifetime history of suicide attempts received treatment. Sociodemographic predictors of treatment utilization in youth with pure suicidal ideation are found in Table 4.^6^ When all factors were considered together in a multivariate analysis, female sex was associated with lower odds of treatment utilization (OR = 0.63, 95% CI 0.48–0.83), as was identifying as Black (OR = 0.57, 95% CI 0.37–0.89) or Hispanic (OR = 0.65, 95% CI 0.43–0.97), and having parents with a high school diploma or GED (OR = 0.45, 95% CI 0.27–0.75). Youth in the lowest quintile for family income had greater odds of receiving treatment (OR = 1.84, 95% CI 1.11–3.05), as did those whose parents were not married (OR = 1.58, 95% CI 1.13–2.21).

**Table 4:** Sociodemographic predictors of psychiatric treatment utilization among children with lifetime history of pure suicidal ideation (unweighted *n* = 1521).

	Any treatment			
	Univariate		Multivariate	
	OR (95% CI)	*p*	OR (95% CI)	*p*
Sex				
Female	0.61 (0.47–0.78)	<0.001	0.63 (0.48–0.83)	<0.01
Male (reference)	1.00		1.00	
Sexual orientation				
Gay or bisexual	1.11 (0.57–2.15)	0.76	1.15 (0.57–2.34)	0.70
Do not understand the question	0.81 (0.61–1.08)	0.15		
Not gay or bisexual (reference)	1.00		1.00	
Race				
Black	0.73 (0.51–1.03)	0.07	0.57 (0.37–0.89)	0.01
Multiracial	0.76 (0.53–1.08)	0.12	0.73 (0.49–1.07)	0.11
Other race	0.61 (0.36–1.01)	0.06	0.65 (0.37–1.13)	0.13
White (reference)	1.00		1.00	
Ethnicity				
Hispanic	0.61 (0.44–0.84)	<0.01	0.65 (0.43–0.97)	0.04
Non-Hispanic (reference)	1.00		1.00	
Family income				
Less than $25,000	1.40 (0.97–2.02)	0.07	1.84 (1.11–3.05)	0.02
$25,000–$49,999	1.36 (0.95–1.94)	0.09	1.52 (0.99-2.33)	0.06
$50,000–$74,999	1.11 (0.78–1.59)	0.56	1.06 (0.71–1.58)	0.78
$75,000 through $99,999	0.86 (0.59–1.27)	0.46	0.96 (0.65–1.43)	0.85
$100,000 and greater (reference)	1.00		1.00	
Parental education				
Less than high school	1.14 (0.41–3.16)	0.81	1.29 (0.35–4.78)	0.71
High school or GED	0.55 (0.37–0.81)	<0.01	0.45 (0.27–0.75)	<0.01
Some college	1.09 (0.79–1.49)	0.61	0.90 (0.62–1.31)	0.59
College graduate (reference)	1.00		1.00	
Parental marital status				
Not married	1.53 (1.20–1.96)	<0.01	1.58 (1.13–2.21)	<0.01
Married (reference)	1.00		1.00	

Multivariate analyses controlled for all other sociodemographic factors. *CI* confidence interval, *GED* General Educational Development, *OR* odds ratio.

Further analyses examined diagnostic predictors of treatment utilization among youth with a history of pure suicidal ideation (Table 5). Multivariate analyses revealed that having one psychiatric diagnosis increased the odds of treatment utilization (OR = 4.89, 95% CI 2.98–8.02), and having two or more diagnoses further increased these odds (OR = 13.84, 95% CI 8.73–21.93). In a multivariate analysis, after controlling for sociodemographic variables, significant predictors included social anxiety (OR = 1.91, 95% CI 1.09–3.34), GAD (OR = 3.08, 95% CI 1.96–4.84), ODD (OR = 2.33, 95% CI 1.65–3.29), and ADHD (OR = 2.11, 95% CI 1.53–2.92).

**Table 5:** Diagnostic predictors of psychiatric treatment utilization among children with lifetime history of pure suicidal ideation (unweighted *n* = 1521).

	Any treatment			
	Univariate		Multivariate	
	OR (95% CI)	*p*	OR (95% CI)	*p*
Any disorder	9.01 (6.04–13.43)	<0.001	9.89 (6.34–15.41)	<0.001
Number of diagnoses				
Single disorder	4.18 (2.65–6.59)	<0.001	4.89 (2.98–8.02)	<0.001
Two or more disorders	12.78 (8.47–19.27)	<0.001	13.84 (8.73–21.93)	<0.001
Disorder type				
MDD	2.14 (1.57–2.93)	<0.001	1.44 (0.97–2.15)	0.07
Any anxiety disorder	3.92 (3.03–5.07)	<0.001		
Separation anxiety	3.61 (2.61–4.99)	<0.001	1.48 (0.95–2.32)	0.09
Social anxiety	3.68 (2.45–5.52)	<0.001	1.91 (1.09–3.34)	0.02
Specific phobia	1.86 (1.44–2.39)	<0.001	1.07 (0.77–1.50)	0.67
GAD	5.63 (3.93–8.08)	<0.001	3.08 (1.96–4.84)	<0.001
OCD	2.23 (1.61–3.10)	<0.001	0.99 (0.63–1.55)	0.96
PTSD	5.54 (3.10–9.90)	<0.001	1.56 (0.74–3.28)	0.24
Any behavioral disorder	4.23 (3.26–5.49)	<0.001		
Conduct disorder	3.10 (2.04–4.72)	<0.001	1.51 (0.85–2.68)	0.16
ODD	4.26 (3.27–5.54)	<0.001	2.33 (1.65–3.29)	<0.001
ADHD	4.35 (3.36–5.63)	<0.001	2.11 (1.53–2.92)	<0.001
Eating disorders	4.32 (1.59–11.71)	<0.01	1.64 (0.42–6.32)	0.47
Psychosis	2.66 (1.06–6.70)	0.04	1.12 (0.28–4.50)	0.88

*CI* confidence interval, *GED* General Educational Development, *OR* odds ratio, *ADHD* attention-deficit hyperactivity disorder, *GAD* generalized anxiety disorder, *MDD* major depressive disorder, *OCD* obsessive compulsive disorder, *ODD* oppositional defiant disorder, *PTSD* post-traumatic stress disorder; *Any anxiety disorder* panic disorder, agoraphobia, separation anxiety disorder, social anxiety disorder, specific phobia, generalized anxiety disorder, obsessive compulsive disorder, or post-traumatic stress disorder; *Any behavioral disorder* conduct disorder or oppositional defiant disorder.

Multivariate analyses separated by blank rows represent separate models, each of which covaried all sociodemographic factors. The first model examined whether having any diagnosis predicted treatment utilization, the second assessed number of diagnoses (none, single disorder, two or more disorders) as a predictor, and the third assessed each disorder as a predictor controlling for all other diagnoses. Only individual diagnoses were included in multivariate models to avoid overlap between individual diagnoses and grouped diagnoses (i.e., any anxiety disorder and any behavioral disorder).

## Discussion

Data from this nationally representative sample indicate that in preadolescent youth, a lifetime history of suicidal ideation is alarmingly common with suicide attempts more rare, but still of critical concern. The high rates of suicidal ideation and even suicide attempts in this preadolescent sample underscore the need to clarify predictors of suicide risk in children, and in particular, to examine predictors that differentiate children who experience a suicide attempt from those with suicidal ideation. The present study offers unique insight into the predictors of pure suicidal ideation and suicide attempts among preadolescent children in the United States.

Relative to males, females exhibited a lower likelihood of suicidal ideation, but sex was not a significant predictor of suicide attempts. Although rates of suicidal ideation and suicide attempts are consistently higher among female adolescents than male peers [7, 30], this does not appear to be the case in preadolescent children. Prior research with youth in this age group has been mixed regarding sex differences in suicidal ideation with some studies finding females to be more likely to experience suicidal ideation than males [29] and other studies documenting higher rates of suicidal ideation among males than females [13]. In terms of sex differences in suicide attempts, our finding that sex was not associated with having made a suicide attempt is consistent with past research suggesting that sex differences in suicide attempts are not apparent in preadolescent children [29, 31] and may not emerge until adolescence [32]. Our study adds new data from a large, nationally representative sample that emphasizes the need to not overlook the possibility of suicidal ideation in young males. For these males, such early onset of suicidal ideation could be an especially important risk factor for future suicidal behavior, and insofar as early onset of psychopathology is associated with a more severe course, it is possible that they may be highly represented in the group of males who eventually die by suicide. Following preadolescent males with suicidal ideation into later waves of ABCD data will be especially important for better understanding if onset of suicidal ideation at this age, when compared to later onset, is indeed associated with an especially poor long-term prognosis in adolescence and adulthood.

Mounting evidence also demonstrates that sexual minority youth are at elevated risk for self-injurious thoughts and behaviors [33, 34]. Our findings extend this literature, showing that these effects are observable in youth as young as 9- and 10-year-old. In this sample, preadolescents identifying as a sexual minority had almost four times greater odds of experiencing suicidal ideation than those identifying as heterosexual. Among preadolescents with a history of suicidal ideation, those identifying as a sexual minority had over two times greater odds of having made a suicide attempt. Our findings highlight the crucial need for early intervention and prevention efforts aimed at sexual minority youth.

Another group in need of intervention and prevention efforts may be youth whose family income is less than $50,000 per year. In our sample, family incomes of less than $25,000 or $25,000–$49,999 were associated with higher odds of youth having made a suicide attempt. These findings align with previous research evaluating socioeconomic correlates of suicide risk in preadolescent youth. Walsh and colleagues, for example, found that among 11- and 12-year-olds, receiving free or reduced-price school lunch was associated with higher odds of suicidal ideation and suicide attempts among youth with suicidal ideation [15]. Lower-income families face frequent stressors, have fewer resources, and face greater barriers in providing high levels of parental monitoring relative to higher-income families, the latter of which was associated with higher risk of both the suicidal ideation and attempts in previous ABCD Study analyses [19].

In terms of race, the lower odds of suicidal ideation that we observed among Black youth are in line with some previous studies [7]. Recent evidence suggests suicide attempts [35] and deaths by suicide [3] are increasing among Black youth, leading to calls from the NIMH for additional research on this population [5]. Although among youth with suicidal ideation, Black youth did not differ in their odds of having attempted suicide relative to White youth, this does not preclude the possibility that the gap between the rates of suicide attempts and death by suicide among White and Black youth, respectively, may be closing. As the ABCD Study continues, future longitudinal research examining predictors of suicide attempts among Black youth in this sample has promise in informing prevention efforts to mitigate this trend.

It also is important to note that multiracial youth had significantly greater odds of experiencing suicidal ideation, but not suicide attempts, relative to White youth and that Hispanic youth had significantly lower odds of experiencing suicidal ideation, but not suicide attempts, relative to non-Hispanic youth. Prior research with adolescents similarly finds that relative to White youth, multiracial youth are at increased risk for suicidal ideation and suicide attempts [36]. In addition, compared with non-Hispanic individuals, Hispanic individuals are at decreased risk for suicidal ideation and suicide attempts, though this gap appears to be narrowing for youth and may have even reversed in recent years [37]. These findings highlight the importance of future research examining the effects of multiple, and at times intersecting, identities. Racial minority groups experience heightened discrimination, prejudice, and stigmatization, which in turn increases stress and negative mental health outcomes [38]. Intersectionality theory [39] emphasizes that these risks may be further amplified when individuals experience multiple minoritized identities, as in the case of some multiracial youth. Consideration of intersecting racial identities is important for advancing our understanding of suicide risk among youth, as well as informing our perspective for implementing systemic changes to lower risk for youth most in need.

In terms of diagnostic predictors of suicidal ideation and suicide attempts, having at least one psychiatric condition was associated with greater odds of lifetime suicidal ideation and the strongest effects were for youth who had two or more disorders, which predicted both the suicidal ideation and suicide attempts. In fact, youth experiencing comorbidity had nearly four times the odds of a lifetime history of suicidal ideation and over two times the odds of attempting suicide among youth with suicidal ideation. Prior evidence suggests that comorbidity is a robust predictor of suicide risk among both adolescents and adults [7, 40], and the present findings extend this pattern to preadolescent youth. Theories of suicide highlight that greater distress or psychological pain [41] may lead to suicide through the desire to escape this pain [42]. The dose-response relationship observed in our findings and in previous literature may be attributed to the fact that comorbidity is associated with greater levels of distress and impairment [40].

Individual diagnoses were less consistent predictors of suicidal ideation and suicide attempts in this age group. Of particular note is the finding that MDD was associated with substantially increased odds of suicidal ideation but not suicide attempts among those youth with suicidal ideation. That MDD was not significantly associated with suicide attempts is notable because suicidal ideation and behavior are most often thought of as occurring in the context of MDD. The current findings suggest that a more nuanced view of this association may be warranted. That MDD was the strongest diagnostic predictor of suicidal ideation is consistent with this perspective; in fact, the confidence interval for MDD did not overlap with those for any other disorder. In contrast, MDD did not differentiate between the youth with suicidal ideation who had and had not made a suicide attempt. This is in line with a recent meta-analysis that found depression to robustly predict suicidal ideation but only weakly predict suicide attempts among individuals with suicidal ideation [43].

Also of note are the findings that conduct disorder, ODD, and ADHD were associated with suicidal ideation but not suicide attempts in multivariate models. Externalizing disorders often are overlooked when evaluating diagnostic factors associated with suicide risk. In adolescence, however, disruptive behavior disorders, including conduct disorder, ODD, and ADHD were associated with suicidal ideation and attempts in nationally representative data [7]. Findings in this sample of preadolescents suggests a potential pathway in which youth with externalizing psychopathology experience suicidal ideation, which only later in development manifests as suicidal behavior. Specifically, these findings collectively suggest that preadolescent youth with MDD or externalizing disorders may be at particular risk for suicidal ideation, but that psychiatric comorbidity could potentially be even more important in understanding which of these youth may go on to act on their suicidal thoughts. Longitudinal research is needed to test this possibility, however.

We found that 34.59% of preadolescent youth with a lifetime history of suicidal ideation and 54.82% of youth with a lifetime history of suicide attempts received psychiatric treatment. These rates are lower than those documented in studies assessing treatment utilization among adolescents with a history of suicidal ideation or behavior (>80%) [7] and indicate that too few suicidal preadolescent youth receive treatment. One factor may be that preadolescent youth are not disclosing suicidal ideation or behavior to their parents/guardians. This is supported by studies finding of low concordance between youth and parent reports of suicidal ideation and suicide attempts [22, 44, 45].

It also is possible that these low rates of treatment utilization are due to expressions of suicidal ideation among preadolescent youth relative to adolescents being viewed as less serious or perhaps even discounted due to beliefs that young children are less likely truly to comprehend suicide or have the means to act on suicidal thoughts [46, 47]. Among youth with suicidal ideation, youth who identified as female, Black, or Hispanic had lower odds of receiving treatment. There exist long standing racial and ethnic disparities in access to healthcare across a host of health conditions; [48–50] our findings suggest that this issue may extend to access to mental health treatment for preadolescent youth experiencing suicidal ideation. The finding of lower treatment utilization among Black youth with suicidal ideation, in particular, adds urgency to the aforementioned recent emphasis by the NIMH to better understand suicide in this age and racial demographic. Youth with suicidal ideation who had any mental health diagnosis, and especially two or more mental health diagnoses, had greater odds of having received mental health treatment. This is in line with research among adolescents [20], which found higher rates of treatment utilization when adolescents were experiencing greater levels of psychopathology. Thus, it may be that comorbid psychopathology, rather than suicidal ideation itself, is a major driver of initiating mental health treatment.

### Strengths and limitations

Strengths of this study include the large population-based sample which allows for meaningful investigation of low base-rate behaviors. This is particularly relevant because such large population-representative studies of preadolescent suicide are exceedingly rare and yet necessary to accurately characterize the scale of this public health concern. Additionally, suicide was assessed using a structured diagnostic interview, which is more robust and less prone to misclassification than the single-item assessment approach used in most large-scale studies [51, 52]. Yet, these findings must be interpreted in the context of several limitations. First, the cross-sectional nature of the data precludes inferences regarding temporality. Future research should examine prospective relationships between the comorbidity and suicide as ABCD follow-up data become available as the study progresses. Second, groups within certain sociodemographics, such as race and sexual orientation, were combined to retain sufficient analytical power, preventing fine-grained examination of between-group differences. This is important as risk for negative mental health outcomes may differ based on one’s specific identity; for example, in a study on suicide rates among lesbian, gay, and bisexual young adults, risk for suicidal ideation and behavior was higher for individuals who identified as bisexual compared with lesbian or gay [53]. Finally, we examined predictors of any mental health treatment utilization. Future research examining types of services utilized, duration of treatment, or effectiveness of treatment received could inform more specific recommendations regarding how best to connect youth experiencing suicidal ideation or behavior with evidence-based care.

### Clinical implications

Results support the critical need for targeted prevention and treatment approaches for suicide in preadolescent children. Indeed, a recent meta-analytic review found current intervention approaches for suicide and self-injury exhibit relatively small effects [54], and these effects were even weaker for child and adolescent samples for which there are far fewer studies than for adults. The current findings shed light on important sociodemographic and diagnostic considerations when developing targeted prevention efforts for both the suicidal ideation and attempts in this age group. Specifically, selective prevention approaches in preadolescent children may benefit from a greater focus on Black and sexual minority youth, youth from low-income families, and youth with multiple psychiatric comorbidities. Data also suggest that targeted approaches may differ for the prevention of suicidal ideation and suicide attempts. In addition to considering whom to target for prevention and treatment efforts, results highlight the need for improving engagement of mental health services in preadolescent youth with histories of suicidal ideation and/or suicide attempts. That preadolescents with suicidal ideation or suicide attempts were found to receive treatment at lower rates than their adolescent counterparts [7] may be due to a lack of recognition among parents and providers of the seriousness of expressions of suicidality in this age group. Educating providers and stakeholders about the clinical importance of preadolescent suicidal thoughts and behaviors, as evidenced in part by the fact that they tend to be accompanied by significant psychiatric comorbidity, may be a necessary first step in improving the identification of at-risk youth. In turn, regular screening for suicidal ideation and suicide attempts in settings where youth are regularly seen (e.g., primary care, school), when paired with referrals to appropriate and accessible mental health services, may help to lower rates of suicidal ideation and behavior among preadolescent youth.

## Supplementary information

Sociodemographic predictors of psychiatric treatment utilization among children with lifetime history of suicidal ideation and/or suicide attempts (unweighted *n* = 1648)

Diagnostic predictors of psychiatric treatment utilization among children with lifetime history of suicidal ideation and/or suicide attempts (unweighted *n* = 1648)

## Data Availability

All ABCD Study data is stored in the NIMH Data Archive Collection #2573.

## Footnotes

1 Janiri and colleagues reported low interinformant agreement between child and parent/guardian reports of suicidal ideation and attempts (Cohen’s κ 0–0.2) in this ABCD dataset [22]. Predictors of suicidal ideation and attempts were largely similar regardless of informant in their study. For this reason and to follow convention [28], child and parent/guardian reported suicidal ideation and attempts were combined in this study to ensure all suicide risk was captured.

2 Categories were less than $25,000, $25,000–$49,999, $50,000–$74,999, $75,000–$99,999, and $100,000 and greater.

3 The small number of unweighted cases of suicidal ideation and suicide attempts for several groups required collapsing these variables for meaningful analyses.

4 “Not sure” responses were not included in analyses.

5 In line with previous studies [29], this approach allows for evaluation of the strength of the association between sociodemographic and diagnostic factors and suicidal ideation unconfounded by the presence of suicide attempts among preadolescents with suicidal ideation.

6 Analyses could not be conducted assessing sociodemographic and diagnostic predictors of treatment utilization among youth with lifetime histories of suicide attempts given that this sample of youth was not sufficiently large for statistical analysis. Therefore, sensitivity analyses also were conducted assessing sociodemographic (see Table S1) and diagnostic (see Table S2) predictors of treatment utilization among youth with suicidal ideation and/or suicide attempts (unweighted *n* = 1648). Results were largely identical.
