# Supplementary material for: Prevalence and correlates of suicidal ideation and suicide attempts in preadolescent children: A US population-based study
Source: Transl Psychiatry. 2021 Sep 22;11:489. doi: 10.1038/s41398-021-01593-3 (PMC8458398; doi:10.1038/s41398-021-01593-3)
Supplement: Supplementary file 1 — Sociodemographic predictors of psychiatric treatment utilization among children with lifetime history of suicidal ideation and/or suicide attempts (unweighted n = 1648) [file 41398_2021_1593_MOESM1_ESM.docx]

| Table S1. Sociodemographic predictors of psychiatric treatment utilization among children with lifetime history of suicidal ideation and/or suicide attempts (unweighted *n* = 1648) | | | | | |
| --- | --- | --- | --- | --- | --- |
|  | | Any treatment | | | |
|  | | Univariate | | Multivariate | |
|  | | OR (95% CI) | *p* | OR (95% CI) | *p* |
| Sex | |  | |  |  |
|  | Female | 0.64 (0.50-0.81) | <.001 | 0.63 (0.49-0.82) | <.01 |
|  | Male (reference) | 1.00 |  | 1.00 |  |
| Sexual orientation | |  | |  |  |
|  | Gay or bisexual | 1.35 (0.74-2.46) | .34 | 1.48 (0.77-2.82) | .24 |
|  | Do not understand the question | 0.85 (0.64-1.11) | .23 | 0.87 (0.65-1.18) | .38 |
|  | Not gay or bisexual (reference) | 1.00 |  | 1.00 |  |
| Race | |  | |  |  |
|  | Black | 0.74 (0.53-1.02) | .07 | 0.58 (0.39-0.87) | <.01 |
|  | Multiracial | 0.82 (0.59-1.15) | .26 | 0.78 (0.53-1.13) | .18 |
|  | Other Race | 0.56 (0.34-0.92) | .02^a^ | 0.59 (0.35-1.00) | .05 |
|  | White (reference) | 1.00 |  | 1.00 |  |
| Ethnicity | |  |  |  |  |
|  | Hispanic | 0.67 (0.50-0.90) | <.01 | 0.75 (0.52-1.09) | .13 |
|  | Non-Hispanic (reference) | 1.00 |  | 1.00 |  |
| Family Income | |  |  |  |  |
|  | Less than $25,000 | 1.35 (0.96-1.90) | .09 | 1.78 (1.10-2.86) | .02 |
|  | $25,000 to $49,999 | 1.42 (1.02-1.97) | .04^a^ | 1.57 (1.05-2.33) | .03 |
|  | $50,000 to $74,999 | 1.10 (0.78-1.55) | .59 | 1.04 (0.71-1.53) | .84 |
|  | $75,000 through $99,999 | 0.86 (0.60-1.25) | .44 | 0.94 (0.64-1.38) | .76 |
|  | $100,000 and greater (reference) | 1.00 |  | 1.00 |  |
| Parental education | |  |  |  |  |
|  | Less than high school | 0.93 (0.36-2.43) | .89 | 1.09 (0.29-4.13) | .90 |
|  | High school or GED | 0.56 (0.39-0.80) | <.01 | 0.46 (0.29-0.73) | <.01 |
|  | Some college | 1.01 (0.75-1.37) | .93 | 0.81 (0.57-1.15) | .24 |
|  | College graduate (reference) | 1.00 |  | 1.00 |  |
| Parental marital status | |  |  |  |  |
|  | Not married | 1.58 (1.25-1.99) | <.001 | 1.63 (1.19-2.23) | <.01 |
|  | Married (reference) | 1.00 |  | 1.00 |  |

^a^Not significant after Benjamini-Hochberg correction applied.

*Note*. Multivariate analyses controlled for all other sociodemographic factors. CI = confidence interval; GED = General Educational Development; OR = odds ratio.
