# Supplementary material for: Prevalence and correlates of suicidal ideation and suicide attempts in preadolescent children: A US population-based study
Source: Transl Psychiatry. 2021 Sep 22;11:489. doi: 10.1038/s41398-021-01593-3 (PMC8458398; doi:10.1038/s41398-021-01593-3)
Supplement: Supplementary file 2 — Diagnostic predictors of psychiatric treatment utilization among children with lifetime history of suicidal ideation and/or suicide attempts (unweighted n = 1648) [file 41398_2021_1593_MOESM2_ESM.docx]

| Table S2. Diagnostic predictors of psychiatric treatment utilization among children with lifetime history of suicidal ideation and/or suicide attempts (unweighted *n* = 1648) | | | | | | | |
| --- | --- | --- | --- | --- | --- | --- | --- |
|  | | | Any treatment | | | | |
|  | | | Univariate | |  | Multivariate | |
|  | | | OR (95% CI) | *p* |  | OR (95% CI) | *p* |
| Any disorder | | | 8.79 (6.05-12.78) | <.001 |  | 9.51 (6.23-14.51) | <.001 |
|  |  | |  |  |  |  |  |
| Number of diagnoses | | | |  |  |  |  |
|  | Single disorder | | 4.01 (2.61-6.15) | <.001 |  | 4.65 (2.90-7.46) | <.001 |
|  | Two or more disorders | | 12.39 (8.43-18.21) | <.001 |  | 13.06 (8.43-20.25) | <.001 |
|  | | |  |  |  |  |  |
| Disorder type | | |  |  |  |  |  |
|  | MDD | | 2.32 (1.72-3.12) | <.001 |  | 1.49 (1.02-2.18) | .04 |
|  | Any anxiety disorder | | 3.83 (3.00-4.90) | <.001 |  |  |  |
|  |  | Separation anxiety | 3.51 (2.58-4.78) | <.001 |  | 1.39 (0.89-2.15) | .15 |
|  |  | Social anxiety | 3.43 (2.35-4.99) | <.001 |  | 1.67 (0.99-2.81) | .05 |
|  |  | Specific phobia | 1.93 (1.52-2.45) | <.001 |  | 1.10 (0.80-1.50) | .57 |
|  |  | GAD | 5.69 (4.02-8.05) | <.001 |  | 2.96 (1.92-4.56) | <.001 |
|  |  | OCD | 2.35 (1.72-3.21) | <.001 |  | 1.02 (0.67-1.57) | .92 |
|  |  | PTSD | 6.42 (3.64-11.32) | <.001 |  | 2.30 (1.01-5.20) | <.05 |
|  | Any behavioral disorder | | 4.58 (3.57-5.88) | <.001 |  |  |  |
|  |  | Conduct disorder | 3.44 (2.32-5.10) | <.001 |  | 1.77 (1.04-3.02) | .04 |
|  |  | ODD | 4.48 (3.48-5.76) | <.001 |  | 2.40 (1.73-3.33) | <.001 |
|  | ADHD | | 4.50 (3.52-5.75) | <.001 |  | 2.08 (1.53-2.84) | <.001 |
|  | Eating disorders | | 5.27 (2.01-13.81) | <.01 |  | 1.74 (0.48-6.27) | .40 |
|  | Psychosis | | 2.46 (1.13-5.35) | .02 |  | 0.90 (0.31-2.63) | .84 |

*Note*. Multivariate analyses separated by double horizontal lines represent separate models, each of which covaried all sociodemographic factors. The first model examined whether having any diagnosis predicted treatment utilization, the second assessed number of diagnoses (none, single disorder, two or more disorders) as a predictor, and the third assessed each disorder as a predictor controlling for all other diagnoses. Only individual diagnoses were included in multivariate models to avoid overlap between individual diagnoses and grouped diagnoses (i.e., any anxiety disorder and any behavioral disorder). CI = confidence interval; GED = General Educational Development; OR = odds ratio; ADHD = attention deficit hyperactivity disorder; GAD = generalized anxiety disorder; MDD = major depressive disorder; OCD = obsessive compulsive disorder; ODD = oppositional defiant disorder; PTSD = post-traumatic stress disorder; Any anxiety disorder = panic disorder, agoraphobia, separation anxiety disorder, social anxiety disorder, specific phobia, generalized anxiety disorder, obsessive compulsive disorder, or post-traumatic stress disorder; Any behavioral disorder = conduct disorder or oppositional defiant disorder.
